# Distinct microbiotas of anatomical gut regions display idiosyncratic seasonal variation in an avian folivore

**DOI:** 10.1186/s42523-019-0002-6

**Published:** 2019-02-05

**Authors:** Sergei V. Drovetski, Michael J. V. O’Mahoney, Kenan O. Matterson, Brian K. Schmidt, Gary R. Graves

**Affiliations:** 1 Laboratories of Analytical Biology, National Museum of Natural History, Smithsonian Institution, Washington, DC 20004 USA; 2 Department of Invertebrate Zoology, National Museum of Natural History, Smithsonian Institution, Washington, DC 20004 USA; 3 Department of Vertebrate Zoology, National Museum of Natural History, Smithsonian Institution, Washington, DC 20004 USA; 4 Center for Macroecology, Evolution and Climate, National Museum of Denmark, University of Copenhagen, DK-2100 Copenhagen Ø, Denmark

**Keywords:** Gut microbiota, Gut regions, Seasonal variation, Sage-grouse, *Centrocercus urophasianus*

## Abstract

**Background:**

Current knowledge about seasonal variation in the gut microbiota of vertebrates is limited to a few studies based on mammalian fecal samples. Seasonal changes in the microbiotas of functionally distinct gut regions remain unexplored. We investigated seasonal variation (summer versus winter) and regionalization of the microbiotas of the crop, ventriculus, duodenum, cecum, and colon of the greater sage-grouse (*Centrocercus urophasianus*), an avian folivore specialized on the toxic foliage of sagebrush (*Artemesia spp*.) in western North America.

**Results:**

We sequenced the V4 region of the 16S rRNA gene on an Illumina MiSeq and obtained 6,639,051 sequences with a median of 50,232 per sample. These sequences were assigned to 457 bacterial and 4 archaeal OTUs. *Firmicutes* (53.0%), *Bacteroidetes* (15.2%), *Actinobacteria* (10.7%), and *Proteobacteria* (10.1%)were the most abundant and diverse phyla. Microbial composition and richness showed significant differences among gut regions and between summer and winter. Gut region explained almost an order of magnitude more variance in our dataset than did season or the gut region × season interaction. The effect of season was uneven among gut regions. Microbiotas of the crop and cecum showed the greatest seasonal differences.

**Conclusions:**

Our data suggest that seasonal differences in gut microbiota reflect seasonal variation in the microbial communities associated with food and water. Strong differentiation and uneven seasonal changes in the composition and richness of the microbiota among functionally distinct gut regions demonstrate the necessity of wider anatomical sampling for studies of composition and dynamics of the gut microbiota.

**Electronic supplementary material:**

The online version of this article (10.1186/s42523-019-0002-6) contains supplementary material, which is available to authorized users.

## Background

The vertebrate gut is colonized by diverse microbial communities [1, 2]. The rapidly growing appreciation of the microbial contribution to digestion, immune function, development, and reproduction in vertebrates is primarily based on studies of humans and a relatively small number of domestic and captive species of mammals and birds [2–4]. Consequently, our knowledge of the factors that affect the assembly and dynamics of the gut micriobiota of wild species is limited [3, 5]. Vertebrate diets often exhibit marked seasonal variation in the composition, abundance, and nutritional quality of food resources. Concomitant seasonal changes in fecal microbiotas have been demonstrated in indigenous humans [6], giant pandas [7], squirrels [8], wood mice [9], macaques [10], and ground squirrels [11]. However, the generality of this pattern has not been investigated in non-mammalian vertebrates.

**Fig. 1:**
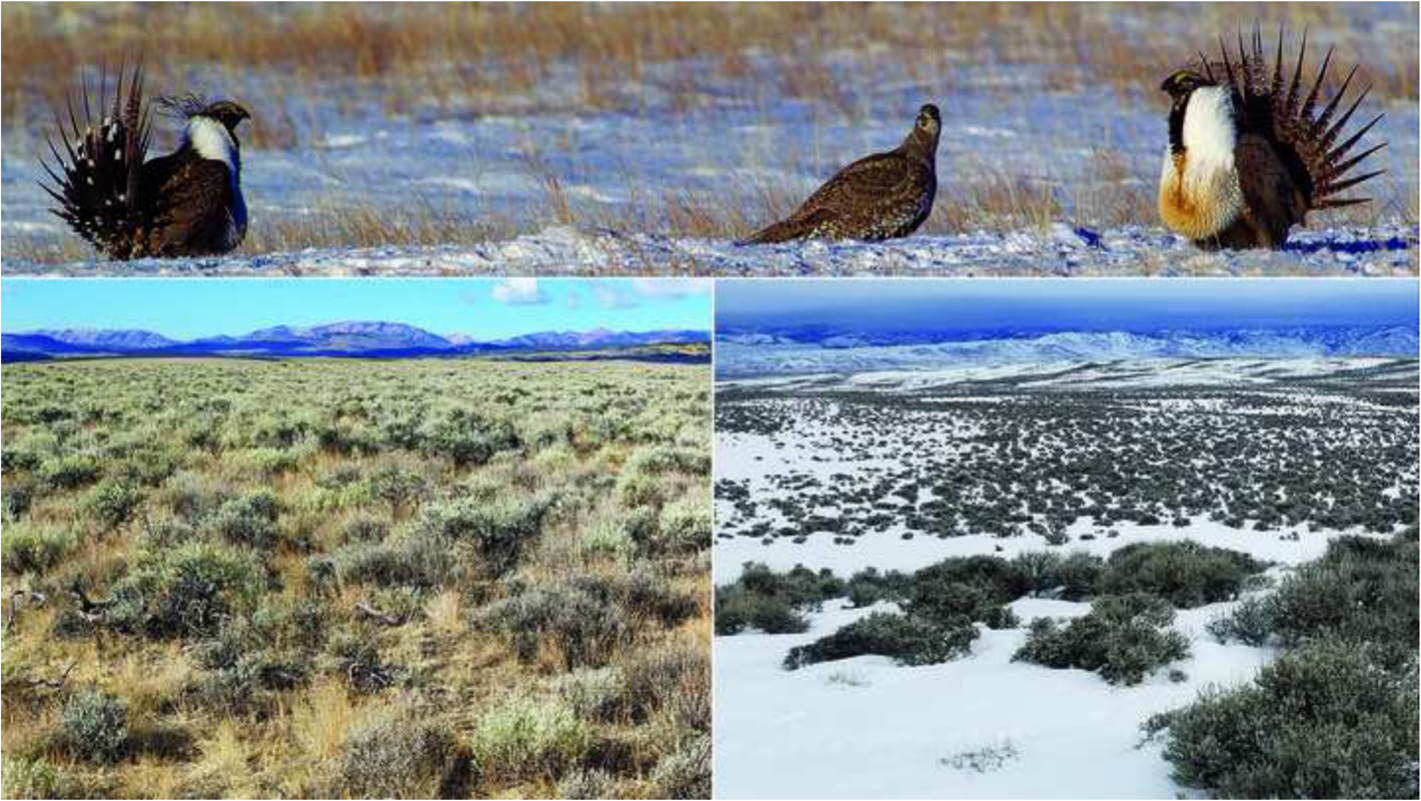
Lekking greater sage-grouse (top panel) and sagebrush habitat in the summer (bottom left panel) and winter (bottom right panel) in Sublette County, Wyoming

Seasonal variation of gut microbiota in vertebrates with specialized diets rich in secondary plant compounds is of particular interest because of the hypothesized functional role of prokaryotes in the degradation and metabolism of dietary toxins [12]. The greater sage-grouse (*Centrocercus urophasianus*; Fig. 1) exhibits one of the most specialized diets among the 10,135 extant species of birds [13, 14]. It feeds predominately on the foliage of chemically-defended sagebrush (*Artemesia* spp.) and its geographic range coincides with the distribution of sagebrush-dominated habitat (Fig. 1) in western North America [15]. Sagebrush foliage is rich in toxic monoterpenes [16–21], phenolics [20, 22], and sesquiterpene lactones [23] that inhibit browsing by ungulates [24–26]. The greater sage-grouse feeds exclusively on evergreen sagebrush foliage in the winter and browses frequently on sagebrush during snow-free months [27]. Kohl et al. [22] recently found that relative to domestic chickens, the cecal microbiota of the greater sage-grouse was enriched in genes of *Bacteroides*, *Eggerthella* and *Clostridium* associated with the metabolism of plant secondary compounds, especially phenolics [28]. This suggests that sage-grouse rely heavily on specialized microbiota to cope with their toxic sagebrush diet. However, Kohl et al. [22] study was limited to cecal samples from three specimens collected during November–December, which precludes any conclusions on seasonal variation in microbiota across multiple gut regions. To date, the seasonal variation of the gut microbiota has yet to be studied in sage-grouse or other wild birds.

In this study we investigated the seasonal variation in the bacterial and archaeal communities (hereafter microbiota) of the gut of the greater sage-grouse. Using next-generation amplicon sequencing of the 16S rRNA gene, we sought to (*i*) characterize the regionalization of microbiota of the crop, ventriculus, duodenum, cecum, and colon within individual sage-grouse; (*ii*) evaluate the seasonal differences in microbiota associated with summer and winter diets in population samples; and (*iii*) determine whether the gut microbiota of females and males differed in composition and richness. Finally, we address the implications of seasonality and anatomical regionalization of gut microbiota on current and future microbiome studies.

## Results

### Diversity and abundance of microbial taxa

Our initial dataset included 8,755,549 sequences obtained from 145 samples (Additional file 1). Five samples of the original 150 were excluded due to poor DNA or PCR yield. Filtering reduced the total number of sequences to 6,639,051. The number of filtered sequences obtained from single samples ranged from 1140 to 102,201 (Additional file 2) with a median of 50,232.

Filtered sequences from the pooled samples of crop, ventriculus, duodenum, cecum, and colon were assigned to 461 OTUs (Additional file 2). Four OTUs were classified as *Archaea* (*Euryarchaeota*; *Thermoplasmata*; *Thermoplasmatales*; [*Thermoplasmatales*]) and 457 as *Bacteria*. All OTUs were taxonomically classified in SILVA v128 16S rRNA gene reference database [29] to order, 93.7% were classified to family, 76.4% to genus, and 3.9% to species.

Ten bacterial phyla were present: *Firmicutes* was the richest (229 OTUs) and the most abundant (53.6% of the total CSS + Log_2_ OTU abundance) phylum. Other common phyla included *Bacteroidetes* (73 OTUs, 15.2% CSS + Log_2_ abundance), *Actinobacteria* (42 OTUs, 10.7%), and *Proteobacteria*, (65 OTUs, 10.1%, Additional file 3). Of 64 bacterial families, *Ruminococcaceae* (*Firmicutes*) was the richest (80 OTUs) and the most abundant (21.0% total CSS + Log_2_ abundance), followed by *Lachnospiraceae* (*Firmicutes*; 42 OTUs, 9.8%), *Bacteroidaceae* (*Bacteroidetes*; 31 OTUs, 8.1%), and *Lactobacillaceae* (*Firmicutes*; 28 OTUs, 6.4%; Additional file 3). Among the 142 bacterial genera, *Ruminococcaceae* UCG-014 was the richest and the most abundant (34 OTUs, 9.4%), followed by *Bacteroides* (31 OTUs, 8.1%) and *Lactobacillus* (28 OTUs, 6.4%, Additional file 3).

### Regionalization of gut microbiota

No single OTU constituted more than 1.1% of all CSS + Log_2_ sequences in the pooled dataset (all gut regions of all birds; Additional file 3). However, most OTUs occurred in multiple gut regions: 308 of 461 OTUs (66.8%) were found in all five gut regions, 111 OTUs (24.1%) were found in four, 28 OTUs (6.1%) in three, 10 OTUs (2.2%) were observed in two gut regions, and only 4 OTUs (0.9%) were detected in a single gut region (Additional file 3).

The connectivity among different gut regions was further demonstrated by the presence of a positive abundance-occupancy relationship between the overall OTU CSS + Log_2_ abundance and the number of samples in which they were detected (N samples containing an OTU = 22.58 + 0.12 × [CSS + Log_2_ OTU count]; adjusted *r*^*2*^ = 0.816, df = 459, *P* < 2.2 × 10^16^). Although the majority of OTUs occurred in all gut regions, 387 OTUs showed significantly different CSS + Log_2_ abundances in different gut regions (Additional file 3).

Our PERMANOVA results indicated that gut region, season of collection, their interaction, latitude and longitude of collection localities had significant effects on microbiota composition (Table 1), whereas sex, body mass and their interactions with other explanatory variables did not. The PERMANOVA *r*^*2*^ value for the effect of gut region (*r*^*2*^ = 0.483) was eight times larger than the values for season (*r*^*2*^ = 0.055), or their interaction (*r*^*2*^ = 0.060), which in turn were six times larger than the values for longitude (*r*^*2*^ = 0.011) and latitude (*r*^*2*^ = 0.010). Gut region, season of collection, and their interaction remained the only significant variables or those with largest effects in PERMANOVAs for individual pairs of gut regions, with the exception of the interaction term in the ventriculus-cecum pair (Additional file 4). Effects of other variables and interactions were much smaller and only significant in some of the pairwise comparisons between gut regions. Season was the only significant variable or had the largest effect in PERMANOVAs for individual gut regions. The effect of the gut region in PERMANOVAs within season was more than 21 times greater than the effect of longitude, which was the only other variable that had a significant effect in both summer and winter samples (Additional file 4).

**Table 1:** PERMANOVA results comparing the effect of the gut region, season, and geographic coordinates on the weighted UniFrac distances among all samples

	df	Sum of Squares	Mean Squares	*F*	*r* ^*2*^	Pr(>F)
Season	1	0.07149	0.07149	19.145	0.055	0.001
Gut region	4	0.62872	0.15718	42.091	0.483	0.001
Longitude	1	0.01449	0.01449	3.879	0.011	0.005
Latitude	1	0.01262	0.01262	3.379	0.010	0.018
Season × Gut Region	4	0.07794	0.01949	5.218	0.060	0.001
Residuals	133	0.49666	0.00373		0.381	
Total	144	1.30192			1.000	

**Fig. 2:**
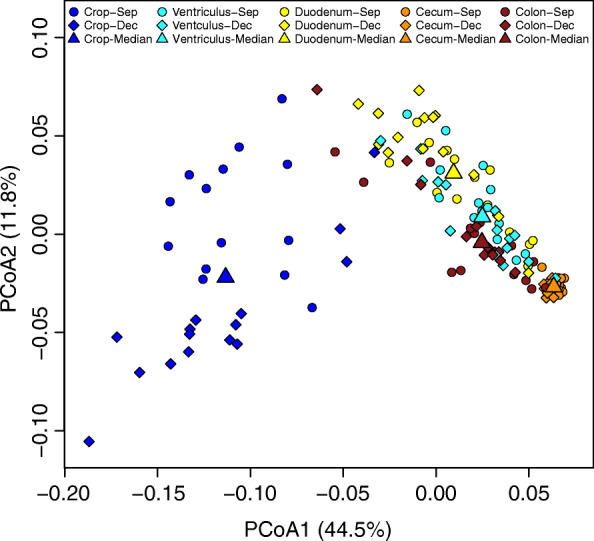
Plot of the principal coordinate analysis of gut microbiota based on weighted UniFrac distances (*n* = 145 samples)

**Fig. 3:**
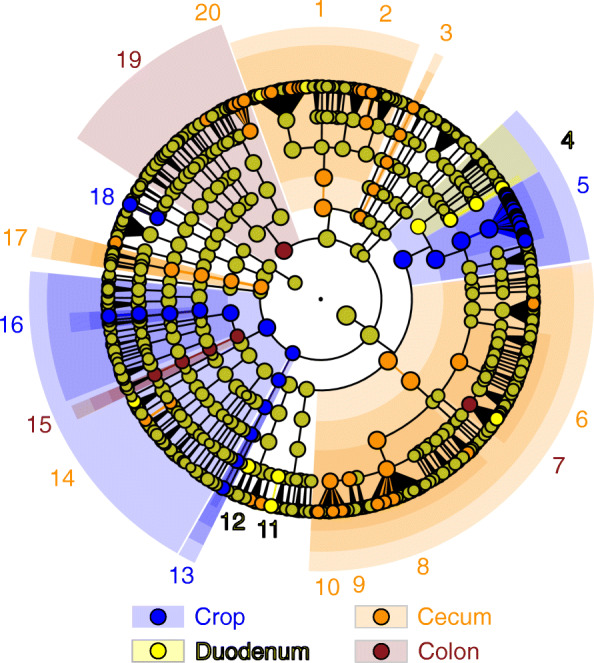
LEfSe results illustrating the lowest-level nested taxa from phylum to genus associated with significant differences in microbiota composition among gut regions: 1 *Bacteroidales*, 2 *Alistipes*, 3 uncl. *Flavobacteriaceae*, 4 *Staphylococcus*, 5 *Lactobacillus*, 6 *Lachnospiraceae*, 7 uncl. *Lachnospiraceae*, 8 *Ruminococcaceae* UCG014, 9 *Eubacterium coprostanoligenes* group, 10 uncl. *Ruminococcaceae*, 11 uncl. *Erysipelotrichaceae*, 12 uncl. *Veillonellaceae*, 13 uncl. *Leptotrichiaceae*, 14 *Oxalobacter*, 15 *Helicobacter*, 16 uncl. *Pasteurellaceae*, 17 *Synergistaceae*, 18 *Mycoplasma*, 19 *Actinobacteria*, 20 uncl. *Coriobacteriaceae*. LDA scores for individual OTUs and nested higher-level taxa are presented in Additional file 5

The clusters of data points for crop and cecum samples did not overlap in the bivariate PCoA plot and displayed limited overlap with those of the ventriculus, duodenum, and colon (Fig. 2). Pairwise PERMANOVA *r*^*2*^ values (Additional file 4) corresponded to the degree of overlap among gut region clusters in the PCoA plot (Fig. 2). Furthermore, LEfSe indicated that crop and cecum microbiotas had larger numbers of distinguishing taxa than other gut regions (Fig. 3, Additional file 5). Significantly higher abundances of the genera *Lactobacillus*, *Mycoplasma*, and unclassified genera of *Pasteurellaceae* and *Leptotrichiaceae* were likely responsible for the observed differences between microbiotas of the crop and other gut regions. The cecum microbiota was distinguished by overrepresentation of two *Clostridia* families: *Ruminococcaceae* and *Lachnospiraceae*, order *Bacteroidales* including *Alistipes*, *Synergistaceae*, *Oxalobacter,* and unclassified genera of *Coriobacteriaceae* and *Flavobacteriaceae*. LEfSe failed to identify any distinguishing taxa from the ventriculus microbiota. The duodenum microbiota was distinguished by an overrepresentation of *Staphylococcus* and unclassified genera of *Erysipelotrichaceae* and *Veillonellaceae*. The colon microbiota was distinguished by higher abundances of the phylum *Actinobacteria*, an unclassified genus of *Lachnospiraceae*, and *Helicobacter*.

**Fig. 4:**
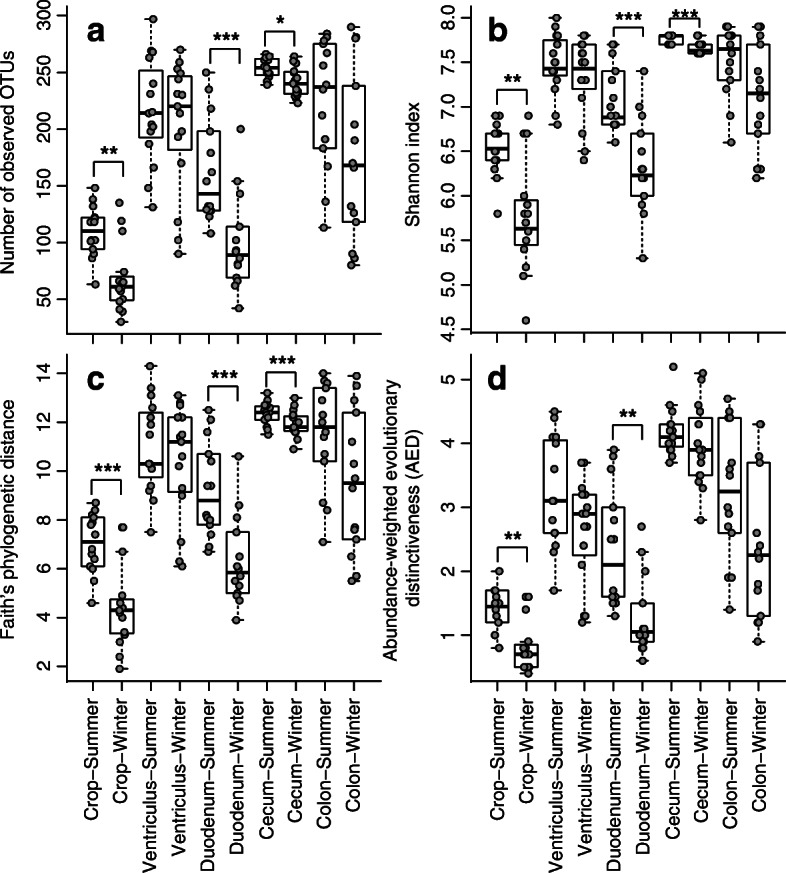
Box plots of microbiota α-diversity indexes for gut regions by season: (**a**) richness (number of observed OTUs), (**b**) abundance and evenness (Shannon index), (**c**) minimum total length of all phylogenetic branches in the community (Faith’s Phylogenetic Diversity index), (**d**) abundance-weighted evolutionary distinctiveness (AED). Wilcoxon Signed Rank test *P*-values identified as follows: *** *P* ≤ 0.001, ** 0.001 < *P* ≤ 0.01, * 0.01 < *P* ≤ 0.05

Gut regions also differed in microbial richness (Fig. 4). The cecum had the richest microbiota (*x̅* = 247.4 ± 12.7 OTUs per sample), followed by ventriculus (*x̅* = 209.1 ± 52.3 OTUs per sample), colon (*x̅* = 198.9 ± 69.4 OTUs per sample), duodenum (*x̅* = 130.7 ± 54.0 OTUs per sample), and crop (*x̅* = 88.0 ± 34.0 OTUs per sample). Linear mixed model regression accounting for the matched design by individual revealed that gut region (*P* < 2.200 × 10^− 16^) and season (*P* = 0.039) were significantly associated with microbiota richness (Fig. 4a) in a model also accounting for sex, longitude, latitude, and body mass (Additional file 6). Neither sex (*P* = 0.936), longitude (*P* = 0.339), latitude (*P* = 0.562), nor body mass (*P* = 0.829) were significantly associated with microbiota richness. Other *α*-diversity indices revealed similar patterns of variation among gut regions and seasons (Fig. 4b-d; Additional file 7).

Gut region was significantly associated with microbiota richness in all subsets restricted to pairs of gut regions or single seasons except the ventriculus - colon pair (Table 2). The season was significantly associated with microbiota richness in all five subsets that include duodenum (four gut region pairs and duodenum) and body mass in three of five subsets that include duodenum (crop – duodenum, duodenum – cecum, and duodenum). The effect of sex, geographic coordinates, or individual was not significantly associated with microbiota richness in any subsets.

**Table 2:** *P-*values for log likelihood tests of linear mixed model regressions for relationships of observed number of OTUs with season, sex, gut region, geographic coordinates, body mass, and accounting for the matched design by individual. Complete outputs of statistical tests are presented in Additional file 6

Subset	Season	Sex	Gut region	Longitude	Latitude	Body mass	Individual
All samples	0.039	0.936	< 0.001	0.339	0.562	0.829	1.000
Crop - Gizzard	0.598	0.265	< 0.001	0.132	0.308	0.434	0.251
Crop - Duodenum	< 0.001	0.194	< 0.001	0.849	0.900	0.003	1.000
Crop - Cecum	0.068	0.627	< 0.001	0.096	0.407	0.601	0.872
Crop - Colon	0.491	0.866	< 0.001	0.401	0.567	0.834	0.978
Ventriculus - Duodenum	0.026	0.898	< 0.001	0.644	0.139	0.511	0.806
Ventriculus - Cecum	0.873	0.123	< 0.001	0.140	0.184	0.136	0.329
Ventriculus - Colon	0.875	0.495	0.464	0.302	0.485	0.192	0.426
Duodenum - Cecum	< 0.001	0.295	< 0.001	0.689	0.697	0.019	0.064
Duodenum - Colon	0.035	0.341	< 0.001	0.884	0.969	0.325	0.616
Cecum - Colon	0.888	0.966	< 0.001	0.593	0.739	0.486	0.785
Crop	0.087	0.962	NA	0.210	0.412	0.363	NA
Ventriculus	0.795	0.232	NA	0.261	0.181	0.213	NA
Duodenum	< 0.001	0.176	NA	0.627	0.676	0.006	NA
Cecum	0.617	0.264	NA	0.242	0.636	0.504	NA
Colon	0.985	0.888	NA	0.721	0.827	0.581	NA
September	NA	0.074	< 0.001	0.314	0.096	0.138	1.000
December	NA	0.084	< 0.001	0.091	0.082	0.091	1.000

**Fig. 5:**
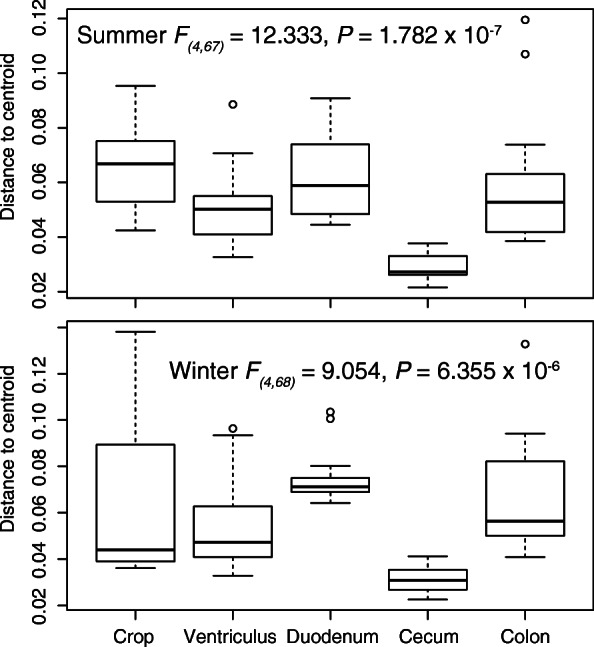
Differences in β-diversity dispersion (average distance to median) among gut regions in summer (top) and winter (bottom). Results of Tukey’s multiple comparison tests are presented in Additional file 8

The cecum samples displayed lower variability than samples from other gut regions. The size of the cecum cluster in the PCoA plot (Fig. 2) was much smaller than the clusters formed by samples from other gut regions. The average distance to centroid (Fig. 5) for the cecal samples (summer: 0.029, winter: 0.031) was significantly lower than the distances (summer: 0.051–0.066, winter: 0.055–0.076) for the other four gut regions during both seasons (Additional file 8). The average distances to centroid for the other four regions did not differ significantly from each other.

### Seasonal variation of the greater sage-grouse microbiota

**Fig. 6:**
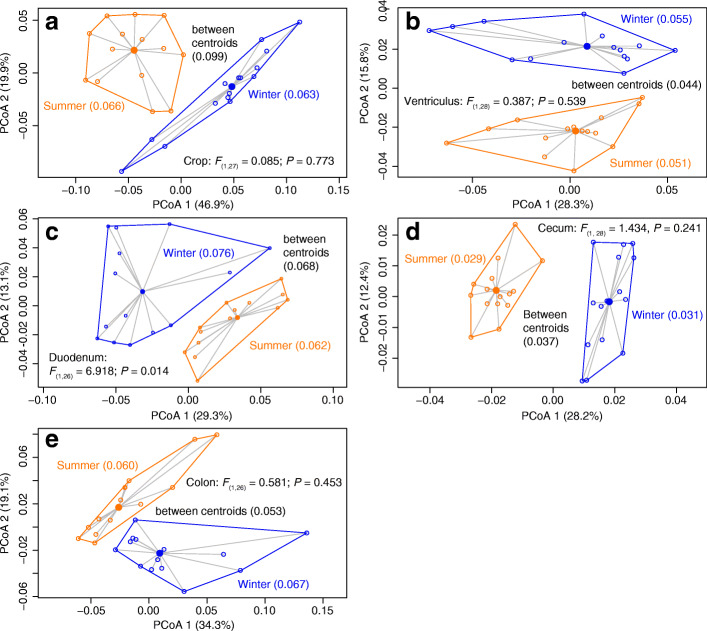
Plots of principal coordinate analyses based on weighted UniFrac distances among individual samples within each gut region: (**a**) crop, (**b**) ventriculus, (**c**) duodenum, (**d**) cecum, (**e**) colon

Microbial richness of the crop, duodenum, and cecum was significantly higher in the summer than in in the winter (Fig. 4). The significant interaction between the gut region and season (Table 1) indicated that season disparately affected the microbiota composition of different gut regions. The strength of seasonal effects (Table 1) was lower in the ventriculus (*r*^*2*^ = 0.141), duodenum (*r*^*2*^ = 0.186), and colon (*r*^*2*^ = 0.172) samples than for those from the cecum (*r*^*2*^ = 0.262) and crop (*r*^*2*^ = 0.321). PCoA plots showed that clusters of summer and winter samples were non-overlapping for all gut regions and average distances to centroid did not differ between seasonal clusters except for the duodenum (Fig. 6). However, the distance between centroids of the season-specific clusters was greater than the intra-cluster mean distances only for the crop and cecum.

LEfSe identified 109 genera whose abundance differed significantly between season in at least one gut region (Additional files 9 and 10). Twenty-eight bacterial genera, likely acquired from food, water, or grit, were relatively more abundant in summer samples, primarily in the crop (22 of 28 genera). Among host-associated bacteria, 36 genera were overrepresented in the summer and 38 in the winter. Four of 36 genera (*Methanogranum*, unclassified *Clostridiales* vadinBB60 group, *Turicibacter*, and unclassified *Synergistaceae*) were overrepresented in all five gut regions in the summer, whereas only two (*Olsenella* and unclassified *Pasteurellaceae*) of 38 genera were overrepresented in all five gut regions in the winter.

## Discussion

Our study provides robust evidence that the greater sage-grouse microbiota exhibits significant regionalization among gut regions. Gut region explained an order of magnitude more variance in microbiota composition than seasonal effects. A significant interaction effect between gut region and season revealed by our analyses reflects idiosyncratic patterns of seasonal changes in microbial communities of different gut regions. Microbiota richness also differed among gut regions and was significantly higher in the summer than in the winter in three of the five sampled gut regions. At the same time, latitude and longitude had limited effects on microbiota richness. We failed to identify any differences between the gut microbiota of females and males or a correlation between OTU richness and body mass of grouse specimens.

The overall composition of the sage-grouse gut microbiota appears similar to the microbiota of other birds [3, 5, 30, 31]. *Firmicutes*, *Bacteroidetes*, *Actinobacteria*, and *Proteobacteria* accounted for 90% of all CSS-normalized and Log_2_-transformed reads and 89% of all OTUs in our dataset. Unfortunately, the lack of standardized protocols in avian microbiota studies [30] and nearly exclusive use of fecal or cloacal samples [3] prevent detailed comparisons of our data with most of previously published studies. The few studies that have sampled multiple gut regions involved poultry or captive birds fed artificial diets: domestic chickens [32–35], captive Attwater’s prairie chickens *Tympanuchus cupido attwateri* [36], and farmed ostriches *Struthio camelus* [37]. A recent study sampled three gut regions from the frozen carcasses of eight species of wild birds (a total of 32 individuals) in Venezuela [38]. Uncertainties about the effects of freezing prior to microbial sampling, sampling protocols, and small sample sizes make it difficult to draw any comparisons with our sage-grouse results.

The best comparative data are provided in a recent study [39] of wild Canada geese (*Branta canadensis*) that employed the same sampling and lab protocols, sequencing platform, and raw data filtering used in our sage-grouse analysis. We found striking similarities in patterns of microbiota richness and regionalization among gut regions in sage-grouse and geese. We observed 461 OTUs in sage-grouse compared to 421 in geese. The cecum had the richest but least variable microbiota in both geese and sage-grouse. The considerably longer retention time of digesta in the cecum, relative to other gut regions [40, 41], permits cecal microbial communities to stabilize and is likely the cause for the reduced variability observed among individuals.

Despite the strong positive abundance-occupancy relationship and low proportion of OTUs restricted to one or two gut regions in sage-grouse and geese, gut regions exhibited significant differences in their microbiota composition. In both studies, the cecum and crop/esophagus had the most distinctive microbiotas. *Pasteurellaceae* and *Leptotrichiaceae* were overrepresented in the crop/esophagus, whereas *Staphylococcaceae* were overrepresented in the duodenum of geese and sage-grouse. *Ruminococcaceae* were overrepresented in the cecum of both species and so were at least some OTUs from *Coriobacteriaceae*, *Bacteroidaceae*, *Prevotellaceae*, and *Rikenellaceae*. Compositional differences among gut regions are likely related to their functional differences [40–43] which impose strong selection on microbiota despite the bidirectional flow of digesta among gut regions [40, 42, 44].

Kohl et al. [22] investigated the cecal microbiota of three individuals of greater sage-grouse with shotgun metagenomic sequencing. The authors provided little information on the taxonomic composition of the cecal microbiotas, but listed *Bacteroides* (19.2% of reads), *Prevotella* (9.6%), and *Clostridium* (9.1%) as the most abundant genera. Our sage-grouse cecal results for these genera (10.5, 0.4, 0.0%, respectively) were significantly different from those reported by Kohl et al. [22] but were similar to the abundances reported for Canada geese (9.5, 2.9, and 0.3%) [39] and Japanese rock ptarmigan (8.1, 2.1, 0.0%) [45]. Furthermore, *Arthrobacter* (*Actinobacteria*: *Micrococcaceae*), which Kohl et al. [22] reported as the main source of genes degrading toxic phenols and catechols in the sage-grouse cecum, was not present in any of our sage-grouse cecal samples, nor were they detected in rock ptarmigan or geese ceca. These discrepancies highlight the need for additional metagenomic analyses of our samples.

Our study appears to be the first to evaluate seasonal microbiota changes in multiple gut regions of a wild vertebrate. Seasonal changes were most pronounced in the crop and cecum microbiotas. The crop is the most anterior of the sampled gut regions and serves as storage organ for consumed foliage before it is released into the ventriculus. We suspect that the pronounced seasonal changes of crop microbiota may be due to marked seasonal variation in the microbial communities of ingested foliage, water, soil, and arthropods as influenced by temperature and snow cover. The paired intestinal ceca are of crucial importance for nutrition in grouse (Aves: Tetraoninae) and are thought to be an adaptation for processing large quantities of poor quality foods [44]. The finely-ground, soluble, and readily digestible forage is shunted to the ceca while large indigestible fragments are rapidly excreted from the gut. The ceca serve as the primary site for the reabsorption of salts and water, and the breakdown of complex carbohydrates (i.e., dietary fiber) and uric acid into volatile fatty acids and ammonia via microbial fermentation [40, 46, 47]. In contrast to other gut regions, ceca size fluctuates substantially in response to seasonal and even short-term changes in diet, increasing concomitantly with the increase of fiber content in forage [40, 46, 47] or when energy demands increase and food quality decreases [44]. Our data suggest that cecal microbiota composition and richness change in response to seasonal diet variation to a greater degree than those of other gut regions distal to the crop.

## Conclusions

Our findings add to a small but rapidly growing body of work that has demonstrated spatial structuring of microbial communities in functionally distinct gut regions of vertebrates: catfish [48], snakes [49], rodents [50], and birds [32–34, 36–39, 51]. Collectively, these analyses suggest that fecal samples alone may be poor predictors of total microbial diversity and abundance in the vertebrate gut. Our study contributes an additional caveat by discovering uneven seasonal changes in microbiota composition and richness among functionally distinct gut regions. These findings together with the independence of region-specific microbiota variation among individuals [39] strongly suggest that future studies of gut microbiota ecology and evolution should sample functionally distinct gut regions in addition to fecal or cloacal samples.

## Methods

### Microbiota sampling

Greater sage-grouse specimens were collected in Wyoming at the end of phenological summer in Sublette County (19–22 September 2016, *n* = 15) and during phenological winter (16–20 December 2016) in Natrona (*n* = 5) and Sublette (*n* = 10) counties (Fig. 1). Detailed data on voucher specimens deposited in the National Museum of Natural History are provided in the Additional file 1.

Greater sage-grouse show marked sexual dimorphism in external measurements and body mass [15]. We measured body mass with a digital scale to the nearest gram before microbiota sampling. Males (*x̅* = 2284 ± 298 g; *n* = 14) included in this study were significantly heavier than the females (*x̅* = 1226 ± 151 g; *n* = 16; Wilcoxon W = 0, *P* = 3.541 × 10^− 6^). Although we did not measure the size or weight of gut regions in individuals, we assumed that luminal volume of different gut regions (e.g., cecum) was correlated with body mass [52].

We sampled five gut regions: crop, ventriculus, duodenum, cecum, and colon [53]. Specimens were put on ice soon after collecting and were kept on ice and processed the same day. We used sterile single-use polyester-tipped applicators with a plastic shaft (Fisher Scientific, Hampton, NH, USA) to sample microbiota from the crop, ventriculus, and colon. Applicators were inserted through a small incision made with a sterile scalpel blade in the crop and ventriculus. For colon sampling, applicators were inserted approximately 30 mm into the colon through the cloaca. Applicators were gently swirled in the lumen and then rubbed on the mucosal lining. Immediately after sampling, applicator ends were snapped off in sterile collection vials, capped, and submerged in liquid nitrogen.

The duodenum and cecum (one side) were sampled by isolating a ~ 25 mm section of the respective gut region with two sterile surgical hemostats. We then injected 1 mL of sterile water (HyPure molecular biology grade water, GE Healthcare Life Sciences, Logan, UT, USA) into the isolated section with a sterile single-use syringe. The water was drawn in and out of the syringe three times to ensure mixing of the intestinal contents. The resulting lavage mixture was immediately transferred into a sterile collection vial, capped, and submerged in liquid nitrogen. All samples were kept in liquid nitrogen until DNA extraction.

### Molecular procedures

Prior to DNA extraction, vials were centrifuged at 2 × 10^4^ G for 20 min and the supernatant was removed with a pipette using sterile filter tips. Total genomic DNA was extracted using the PowerSoil DNA Isolation Kit (Mo Bio Laboratories, Carlsbad, CA, USA) and cleaned with the PowerClean Pro DNA Clean-Up Kit (Mo Bio Laboratories, Carlsbad, CA, USA) to remove PCR inhibitors.

We amplified and sequenced the V4 region of the 16S rRNA gene (252–254 bp) from *Bacteria* and *Archaea* using primers 515FB and 806RB [54], following procedures outlined in the Earth Microbiome Project 16S protocol [54, 55]. Detailed description of PCR conditions, library preparation, and sequencing on an Illumina MiSeq (Illumina, Inc., San Diego, CA, USA) are provided in Drovetski et al. [39].

### Illumina data processing

Raw Illumina data processing included joining of forward and reverse reads (join_paired_ends.py), demultiplexing (split_libraries_fastq.py), and quality filtering (Phred score Q ≥ 30, max barcode error = 0, min length = 200 bp) using the Quantitative Insights Into Microbial Ecology (QIIME) pipeline v1.9.0 [56].

We used UCHIME [57] to identify and remove chimeric sequences against the ChimeraSlayer reference database (version ‘microbiomeutil-r20110519’). The remaining sequences were aligned in PyNAST [58] and taxonomically classified using Bayesian RDP Classifier [59] trained with the SILVA v128 16S rRNA gene reference database [29]. Aligned sequences were clustered into operational taxonomic units (OTUs) at 3% divergence [29, 60], GenBank accession numbers of sequences that matched OTUs in our dataset were retrieved from the SILVA v128 database. We classified OTUs as host-associated or likely obtained through environmental sources (e.g., water, soil, plants, arthropods, etc.) from ‘isolation source’ metadata in GenBank entries and references for accession numbers and their closest matches (99% identity).

Singleton sequences and OTUs with an overall relative abundance < 0.01% were filtered out to reduce the likelihood of sequence artifacts affecting downstream diversity metrics [61]. We also excluded OTUs classified as *Mitochondria*, *Eukaryota*, *Chloroplast*, and those of unknown origin. We constructed an UPGMA tree of remaining OTU representative sequences in Geneious 11.1.4 (Biomatters Ltd., Auckland, New Zealand). We cumulative sum scaled [62] and Log_2_-transformed OTU abundances (CSS + Log_2_ OTU abundances) to account for variation in sequencing depth among samples [63] and non-normal distribution of abundances among OTUs in Calypso 8.10 [64]. All statistical analyses were based on CSS + Log_2_ OTU abundances.

### Data analyses

We constructed rarefaction plots (Additional file 12) with R package Vegan 2.4–4 [65] to ensure our sequencing depth was sufficient and OTU accumulation curves in Microsoft Excel v14.7.7 to evaluate sampling effort of individual birds (Additional file 12). We used QIIME for α and β-diversity analyses. We calculated all available *α*-diversity indexes (alpha_diversity.py). We also calculated abundance-weighted evolutionary distinctiveness (AED) [66] in the R package BAT (https://cran.r-project.org/web/packages/BAT/). Differences in OTU abundances among gut regions were tested using non-parametric Kruskal-Wallis tests (group_significance.py).

We calculated weighted UniFrac distances [67] among individual samples and conducted PCoA (beta_diversity_through_plots.py) to compare microbiota composition. Weighted UniFrac distances were also used in the Permutational Multivariate Analysis of Variance (PERMANOVA) [68] implemented in R package Vegan 2.4–4 [65] to test for the effect of the gut region (crop, ventriculus, duodenum, cecum, and colon), season (summer and winter), sex (male and female), geographic coordinates, body mass, and their interactions on microbiota variation among samples. Effect of the same variables on microbiota richness was evaluated using linear mixed model regression accounting for the matched design by individual grouse specimen. Their significance was determined by log likelihood tests.We used R version 3.3.3 (http://www.R-project.org) to generate Principal Coordinate Analysis (PCoA) and box plots. We modeled the abundance-occupancy relationship [69, 70] by regressing prevalence of OTUs on their total abundance. Paired Wilcoxon Signed Rank tests were used to compare richness among gut regions, body mass of males and females, and *F*-tests were used to compare variance of the PCoA scores among gut regions. Radar plots were made in Microsoft Excel v14.7.7.

We used the Linear Discriminant Analysis effect size (LEfSe) algorithm implemented in the LEfSe software package [71] to identify microbial taxa that best explained microbiota differences among gut regions and seasons. LEfSe consists of three consecutive steps: (*i*) Kruskal-Wallis tests to identify taxa with significantly different abundances among groups, (*ii*) a pairwise Wilcoxon Signed Rank test to select the subsets of taxa overrepresented in only a single group, and (*iii*) Linear Discriminant Analysis to estimate the effect size of each taxon.

## Additional files

Additional file 1: Voucher specimens deposited at the National Museum of Natural History (Smithsonian Institution), sample IDs for different gut regions, sex, age, and locality data for birds used in this study. (XLSX 57 kb)

Additional file 2: Distribution of raw OTU counts among gut regions and individual samples. (XLSX 323 kb)

Additional file 3: Taxonomy and distribution of CSS-normalized and Log_2_-transformed OTU counts among gut regions and individual samples. (XLSX 491 kb)

Additional file 4: Results of PERMANOVAs for the effect of the season, sex, gut region, longitude, latitude, body mass, and their interactions on the weighted UniFrac distances among samples in different subsets of data. (XLSX 58 kb)

Additional file 5: LEfSe scores and FDR-corrected *P*-values for microbial taxa most likely responsible for differences among gut regions. (XLSX 15 kb)

Additional file 6: Results of the log likelihood tests of the mixed linear models for the association between OTU richness and season, sex, gut region, longitude, latitude, body mass, and individual in different subsets of data. (XLSX 21 kb)

Additional file 7: Alpha diversity indices, season, sex, gut region, longitude, latitude, and body mass for individual samples. (XLSX 68 kb)

Additional file 8: Results of Tukey’s multiple comparison tests for β-diversity dispersion (differences in average distance to multidimensional median) among gut regions in different seasons. (PDF 44 kb)

Additional file 9: Radar plots summarizing LEfSe results at the genus level. Each radial line represents a genus significantly overrepresented in summer (top) and winter (bottom) microbiota. The scale and distance from the center of the plot to a data point represents the Log_10_ (LDA score). Point colors represent different gut regions according to the legend. Blue font of the generic names indicates environmental genera. Gray font shows genera with different seasonal assignments in different gut regions. Bold black font identifies genera with consistent seasonal differences in all five gut regions. Regular black font identifies genera with seasonal abundance differences in at least a single gut region. LDA scores for individual OTUs and nested higher-level taxa are presented in Additional file 10. (PDF 369 kb)

Additional file 10: LEfSe scores and FDR corrected *P*-values for microbial taxa most likely responsible for differences between summer and winter in each gut region. (XLSX 66 kb)

Additional file 11: Rarefaction plots for individual samples in each gut region. (TIF 2081 kb)

Additional file 12: OTU accumulation plots. Individual birds were added in the order they were sampled. (PDF 124 kb)

## Abbreviations

AED: Abundance-weighted evolutionary distinctiveness
bp: Base pairs
CSS: Cumulative sum scaled
DNA: Deoxyribonucleic acid
G: Gravity
LEfSe: Linear discriminant analysis effect size
OTU: Operational taxonomic unit
PCoA: Principal coordinates analysis
PCR: Polymerase chain reaction
PERMANOVA: Permutational multivariate analysis of variance
rRNA: Ribosomal ribonucleic acid
UniFrac: Unique fraction

## References

[1] Fraune S, Bosch TCG (2010). Why bacteria matter in animal development and evolution. BioEssays.

[2] Hanning I, Diaz-Sanchez S (2015). The functionality of the gastrointestinal microbiome in non-human animals. Microbiome..

[3] Colston TJ, Jackson CR (2016). Microbiome evolution along divergent branches of the vertebrate tree of life: what is known and unknown. Mol Ecol.

[4] Hird SM (2017). Evolutionary biology needs wild microbiomes. Front Microbiol.

[5] Kohl KD (2012). Diversity and function of the avian gut microbiota. J Comp Physiol B.

[6] Smits SA, Leach J, Sonnenburg ED, Gonzalez CG, Lichtman JS, Reid G (2017). Seasonal cycling in the gut microbiome of the Hadza hunter-gatherers of Tanzania. Science.

[7] Wu Q, Wang X, Ding Y, Hu Y, Nie Y, Wei W, et al. Seasonal variation in nutrient utilization shapes gut microbiome structure and function in wild giant pandas. Proc R Soc B Biol Sci. 2017;284(1862):20170955. 10.1098/rspb.2017.0955 PMC5597826 28904137

[8] Ren T, Boutin S, Humphries MM, Dantzer B, Gorrell JC, Coltman DW (2017). Seasonal, spatial, and maternal effects on gut microbiome in wild red squirrels. Microbiome.

[9] Maurice CF, Cl Knowles S, Ladau J, Pollard KS, Fenton A, Pedersen AB (2015). Marked seasonal variation in the wild mouse gut microbiota. ISME J..

[10] Sun B, Wang X, Bernstein S, Huffman MA, Xia D-P, Gu Z (2016). Marked variation between winter and spring gut microbiota in free-ranging Tibetan Macaques (*Macaca thibetana)*. Sci Rep.

[11] Carey HV, Walters WA, Knight R (2013). Seasonal restructuring of the ground squirrel gut microbiota over the annual hibernation cycle. Am J Physiol Regul Integr Comp Physiol.

[12] Hammer TJ, Bowers MD (2015). Gut microbes may facilitate insect herbivory of chemically defended plants. Oecologia.

[13] Dickinson EC, Christidis L (2014). The Howard and Moore complete checklist of the birds of the world fourth edition, volume 2: passerines.

[14] Dickinson EC, Remsen JV (2013). The Howard and Moore complete checklist of the birds of the world fourth edition, volume 1: non-passerines.

[15] Schroeder MA, Young JR, Braun CE, Rodewald PG (1999). Greater sage-grouse (*Centrocercus urophasianus*), version 2.0. The birds of North America.

[16] Shafizadeh F, Bhadane NR, Kelsey RG (1974). Sesquiterpene lactones of sagebrush: constituents of *Artemisia tripartita*. Phytochemistry.

[17] Welch BL, McArthur ED (1981). Variation of monoterpenoid content among subspecies and accessions of *Artemisia tridentata* grown in a uniform garden. J Range Manag.

[18] Kelsey RG (1982). The chemical constituents of sagebrush foliage and their isolation. —. J Range Manag.

[19] Wilt FM, Miller GC (1992). Seasonal variation of coumarin and flavonoid concentrations in persistent leaves of Wyoming big sagebrush (*Artemisia tridentata ssp. wyomingensis*: Asteraceae). Biochem Syst Ecol.

[20] Wilt FM, Geddes JD, Tamma RV, Miller GC, Everett RL (1992). Interspecific variation of phenolic concentrations in persistent leaves among six taxa from subgenus Tridentatae of *Artemisia* (Asteraceae). Biochem Syst Ecol.

[21] Kohl KD, Pitman E, Robb BC, Connelly JW, Dearing MD, Forbey JS (2015). Monoterpenes as inhibitors of digestive enzymes and counter-adaptations in a specialist avian herbivore. J Comp Physiol B.

[22] Kohl KD, Connelly JW, Dearing MD, Forbey JS. Microbial detoxification in the gut of a specialist avian herbivore, the Greater Sage-Grouse. FEMS Microbiol Lett. 2016;363(14). 10.1093/femsle/fnw144 27242374

[23] Kelsey RG, Morris MS, Shafizadeh F (1976). The use of sesquiterpene lactones as taxonomic markers in the shrubby species of *Artemisia* (section Tridentatae) in Montana. J Range Manag.

[24] Olsen FW, Hansen RM (1977). Food relations of wild free-roaming horses to livestock and big game, Red Desert, Wyoming. J Range Manag.

[25] Johnson MK (1979). Foods of primary consumers on cold desert shrub-steppe of southcentral Idaho. J Range Manag.

[26] Hanley TA, Kathleen AH (1982). Food resource partitioning by sympatric ungulates on Great Basin rangeland. J Range Manag.

[27] Wallestad R, Eng RL (1975). Foods of adult sage grouse in Central Montana. J Wildl Manag.

[28] Selma MV, Espín JC, Tomás-Barberán FA (2009). Interaction between Phenolics and gut microbiota: role in human health. J Agric Food Chem.

[29] Pruesse E, Quast C, Knittel K, Fuchs BM, Ludwig W, Peplies J (2007). SILVA: a comprehensive online resource for quality checked and aligned ribosomal RNA sequence data compatible with ARB. Nucleic Acids Res.

[30] Waite D, Taylor M (2015). Exploring the avian gut microbiota: current trends and future directions. Front Microbiol.

[31] Waite DW, Taylor MW (2014). Characterizing the avian gut microbiota: membership, driving influences, and potential function. Front Microbiol.

[32] Wang L, Lilburn M, Yu Z (2016). Intestinal microbiota of broiler chickens as affected by litter management regimens. Front Microbiol.

[33] Choi JH, Kim GB, Cha CJ (2014). Spatial heterogeneity and stability of bacterial community in the gastrointestinal tracts of broiler chickens. Poult Sci.

[34] Xiao Y, Xiang Y, Zhou W, Chen J, Li K, Yang H (2017). Microbial community mapping in intestinal tract of broiler chicken. Poult Sci.

[35] Lu J, Idris U, Harmon B, Hofacre C, Maurer JJ, Lee MD (2003). Diversity and succession of the intestinal bacterial community of the maturing broiler chicken. Appl Environ Microbiol.

[36] Zhang Y, Simon SE, Johnson JA, Allen MS. Spatial microbial composition along the gastrointestinal tract of captive Attwater’s prairie chicken. Microb Ecol. 2016;73(4):966–977. 10.1007/s00248-016-0870-1 27752719

[37] Videvall E, Strandh M, Engelbrecht A, Cloete S, Cornwallis CK (2018). Measuring the gut microbiome in birds: comparison of faecal and cloacal sampling. Mol Ecol Resour.

[38] García-Amado MA, Shin H, Sanz V, Lentino M, Martínez LM, Contreras M (2018). Comparison of gizzard and intestinal microbiota of wild neotropical birds. PLoS One.

[39] Drovetski SV, O’Mahoney M, Ransome EJ, Matterson KO, Lim HC, Chesser RT (2018). Spatial organization of the gastrointestinal microbiota in urban Canada geese. Sci Rep.

[40] Svihus B (2014). Function of the digestive system. The J Applied Poultry Res.

[41] Roto SM, Rubinelli PM, Ricke SC (2015). An introduction to the avian gut microbiota and the effects of yeast-based prebiotic-type compounds as potential feed additives. Frontiers in Veterinary Sci.

[42] Duke GE (1997). Gastrointestinal physiology and nutrition in wild birds. Proc Nutr Soc.

[43] Pan D, Yu Z (2014). Intestinal microbiome of poultry and its interaction with host and diet. Gut Microbes.

[44] Remington TE (1989). Why do grouse have ceca? A test of the fiber digestion theory. J Exp Zool.

[45] Ushida K, Segawa T, Tsuchida S, Murata K (2016). Cecal bacterial communities in wild Japanese rock ptarmigans and captive Svalbard rock ptarmigans. J Vet Med Sci.

[46] Svihus B, Choct M, Classen HL (2013). Function and nutritional roles of the avian caeca: a review. World’s Poultry Sci J.

[47] Clench MH, Mathias JR (1995). The avian cecum: a review. The Wilson Bulletin.

[48] McDonald R, Schreier HJ, Watts JEM (2012). Phylogenetic analysis of microbial communities in different regions of the gastrointestinal tract in *Panaque nigrolineatus*, a wood-eating fish. PLoS One.

[49] Colston TJ, Noonan BP, Jackson CR (2015). Phylogenetic analysis of bacterial communities in different regions of the gastrointestinal tract of *Agkistrodon piscivorus*, the cottonmouth snake. PLoS One.

[50] Kohl KD, Dearing MD, Bordenstein SR (2018). Microbial communities exhibit host species distinguishability and phylosymbiosis along the length of the gastrointestinal tract. Mol Ecol.

[51] Han GG, Kim EB, Lee J, Lee J-Y, Jin G, Park J (2016). Relationship between the microbiota in different sections of the gastrointestinal tract, and the body weight of broiler chickens. Springerplus.

[52] Franz R, Hummel J, Kienzle E, Kölle P, Gunga H-C, Clauss M (2009). Allometry of visceral organs in living amniotes and its implications for sauropod dinosaurs. Proc R Soc B Biol Sci.

[53] McLelland J. Apparatus digestorius [systema alimentarium]. In: Baumel, JJ, King, AS, Breazile, JE, Evans, HE, and Vanden Berge, JC. (eds). Handbook of avian anatomy: nomina anatomica avium. Publications of the Nuttall Ornithological Club (USA). no 23. Second edition. Cambridge: The Nuttall Ornithological Club; 1993. p. 301–327.

[54] Caporaso JG, Lauber CL, Walters WA, Berg-Lyons D, Huntley J, Fierer N (2012). Ultra-high-throughput microbial community analysis on the Illumina HiSeq and MiSeq platforms. ISME J.

[55] EMP. Earth Microbiome Project: 16S Illumina amplicon protocol 2017 [cited 2017 Accessed 17 May]. Available from: http://press.igsb.anl.gov/earthmicrobiome/protocols-and-standards/16s/.

[56] Caporaso JG (2010). Qime allows analysis of high-throughput community sequencing data. Nat Methods.

[57] Edgar RC (2010). Search and clustering orders of magnitude faster than BLAST. Bioinformatics.

[58] Caporaso JG, Bittinger K, Bushman FD, DeSantis TZ, Andersen GL, Knight R (2010). PyNAST: a flexible tool for aligning sequences to a template alignment. Bioinformatics.

[59] Wang Q, Garrity GM, Tiedje JM, Cole JR. Naive Bayesian classifier for rapid assignment of rRNA sequences into the new bacterial taxonomy. Appl Environ Microbiol. 2007;73. 10.1128/AEM.00062-07 PMC1950982 17586664

[60] Quast C, Pruesse E, Yilmaz P, Gerken J, Schweer T, Yarza P (2013). The SILVA ribosomal RNA gene database project: improved data processing and web-based tools. Nucleic Acids Res.

[61] Bokulich NA, Subramanian S, Faith JJ, Gevers D, Gordon JI, Knight R (2013). Quality-filtering vastly improves diversity estimates from Illumina amplicon sequencing. Nat Meth.

[62] Paulson JN, Stine OC, Bravo HC, Pop M (2013). Robust methods for differential abundance analysis in marker gene surveys. Nat Methods.

[63] McMurdie PJ, Holmes S (2014). Waste not, want not: why rarefying microbiome data is inadmissible. PLoS Comput Biol.

[64] Zakrzewski M, Proietti C, Ellis JJ, Hasan S, Brion M-J, Berger B (2017). Calypso: a user-friendly web-server for mining and visualizing microbiome–environment interactions. Bioinformatics.

[65] Oksanen J, Blanchet FG, Friendly M, Kindt R, Legendre P, McGlinn D, et al. vegan: community ecology package. R package version 2.4–3. 2017. Available from: https://CRAN.R-project.org/package=vegan.

[66] Cadotte MW, Jonathan Davies T, Regetz J, Kembel SW, Cleland E, Oakley TH (2009). Phylogenetic diversity metrics for ecological communities: integrating species richness, abundance and evolutionary history. Ecol Lett.

[67] Lozupone CA, Hamady M, Kelley ST, Knight R (2007). Quantitative and qualitative β diversity measures lead to different insights into factors that structure microbial communities. Appl Environ Microbiol.

[68] Anderson MJ (2001). A new method for non-parametric multivariate analysis of variance. Austral Ecol.

[69] Gaston KJ, Blackburn TM, Greenwood JJD, Gregory RD, Quinn RM, Lawton JH (2000). Abundance–occupancy relationships. J Appl Ecol.

[70] Gaston KJ (1996). The multiple forms of the interspecific abundance-distribution relationship. Oikos.

[71] Segata N, Izard J, Waldron L, Gevers D, Miropolsky L, Garrett WS (2011). Metagenomic biomarker discovery and explanation. Genome Biol.

