# Supplementary material for: Distinct microbiotas of anatomical gut regions display idiosyncratic seasonal variation in an avian folivore
Source: Anim Microbiome. 2019 Feb 5;1:2. doi: 10.1186/s42523-019-0002-6 (PMC7803122; doi:10.1186/s42523-019-0002-6)
Supplement: Supplementary file 8 — Results of Tukey’s multiple comparison tests for β-diversity dispersion (differences in average distance to multidimensional median) among gut regions in different seasons. (PDF 44 kb) [file 42523_2019_2_MOESM8_ESM.pdf]

Tukey multiple comparisons of means
95% family-wise confidence level

Summer	Crop	Gizzard	Duodenum	Cecum	Colon
Average distance to median	0.066	0.051	0.062	0.029	0.060

Summer	Differece	Lower	Upper	*P* adjusted
Gizzard-Crop	-0.015	-0.032	0.002	0.119
Duodenum-Crop	-0.004	-0.021	0.013	0.971
Cecum-Crop	-0.037	-0.054	-0.020	5.000E-07
Colon-Crop	-0.006	-0.024	0.011	0.832
Duodenum-Gizzard	0.011	-0.006	0.028	0.382
Cecum-Gizzard	-0.022	-0.039	-0.006	0.003
Colon-Gizzard	0.008	-0.009	0.025	0.649
Cecum-Duodenum	-0.033	-0.050	-0.016	6.500E-06
Colon-Duodenum	-0.003	-0.020	0.015	0.993
Colon-Cecum	0.031	0.014	0.048	3.380E-05

Winter	Crop	Gizzard	Duodenum	Cecum	Colon
Average distance to median	0.063	0.055	0.076	0.031	0.067

Winter	Differece	Lower	Upper	*P* adjusted
Gizzard-Crop	-0.008	-0.030	0.014	0.856
Duodenum-Crop	0.012	-0.010	0.035	0.531
Cecum-Crop	-0.032	-0.054	-0.010	0.001
Colon-Crop	0.004	-0.019	0.026	0.990
Duodenum-Gizzard	0.020	-0.002	0.043	0.095
Cecum-Gizzard	-0.024	-0.046	-0.002	0.024
Colon-Gizzard	0.012	-0.011	0.034	0.603
Cecum-Duodenum	-0.044	-0.067	-0.022	4.800E-06
Colon-Duodenum	-0.009	-0.031	0.014	0.820
Colon-Cecum	0.036	0.013	0.058	2.877E-04
