# Supplementary figures and images for: Distinct microbiotas of anatomical gut regions display idiosyncratic seasonal variation in an avian folivore
Source: Anim Microbiome. 2019 Feb 5;1:2. doi: 10.1186/s42523-019-0002-6 (PMC7803122; doi:10.1186/s42523-019-0002-6)

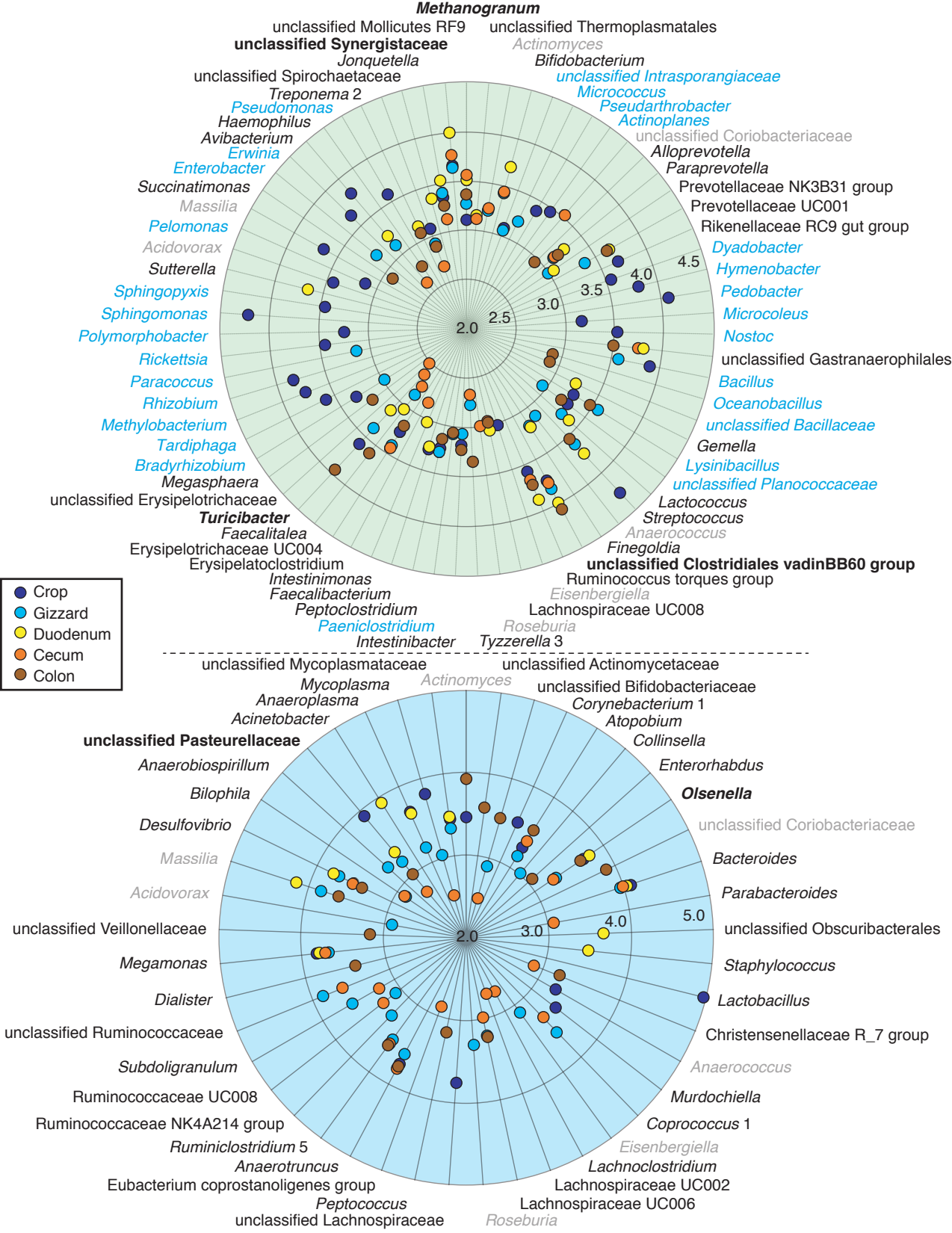

Supplement: Supplementary file 9 — Radar plots summarizing LEfSe results at the genus level. Each radial line represents a genus significantly overrepresented in summer (top) and winter (bottom) microbiota. The scale and distance from the center of the plot to a data point represents the Log10 (LDA score). Point colors represent different gut regions according to the legend. Blue font of the generic names indicates environmental genera. Gray font shows genera with different seasonal assignments in different gut regions. Bold black font identifies genera with consistent seasonal differences in all five gut regions. Regular black font identifies genera with seasonal abundance differences in at least a single gut region. LDA scores for individual OTUs and nested higher-level taxa are presented in Additional file 10. (PDF 369 kb) [file 42523_2019_2_MOESM9_ESM.pdf]

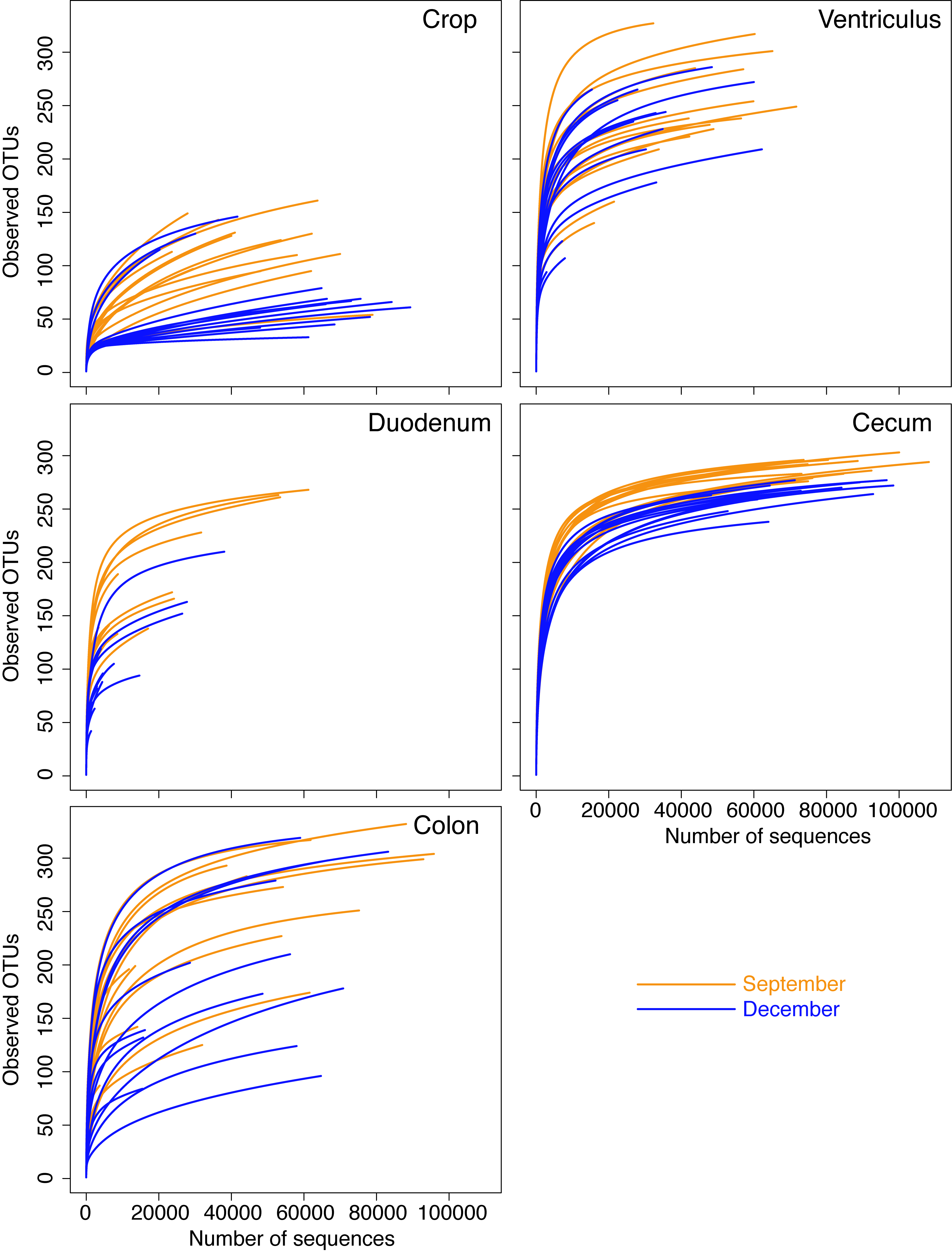

Supplement: Supplementary file 11 — Rarefaction plots for individual samples in each gut region. (TIF 2081 kb) [file 42523_2019_2_MOESM11_ESM.tif]

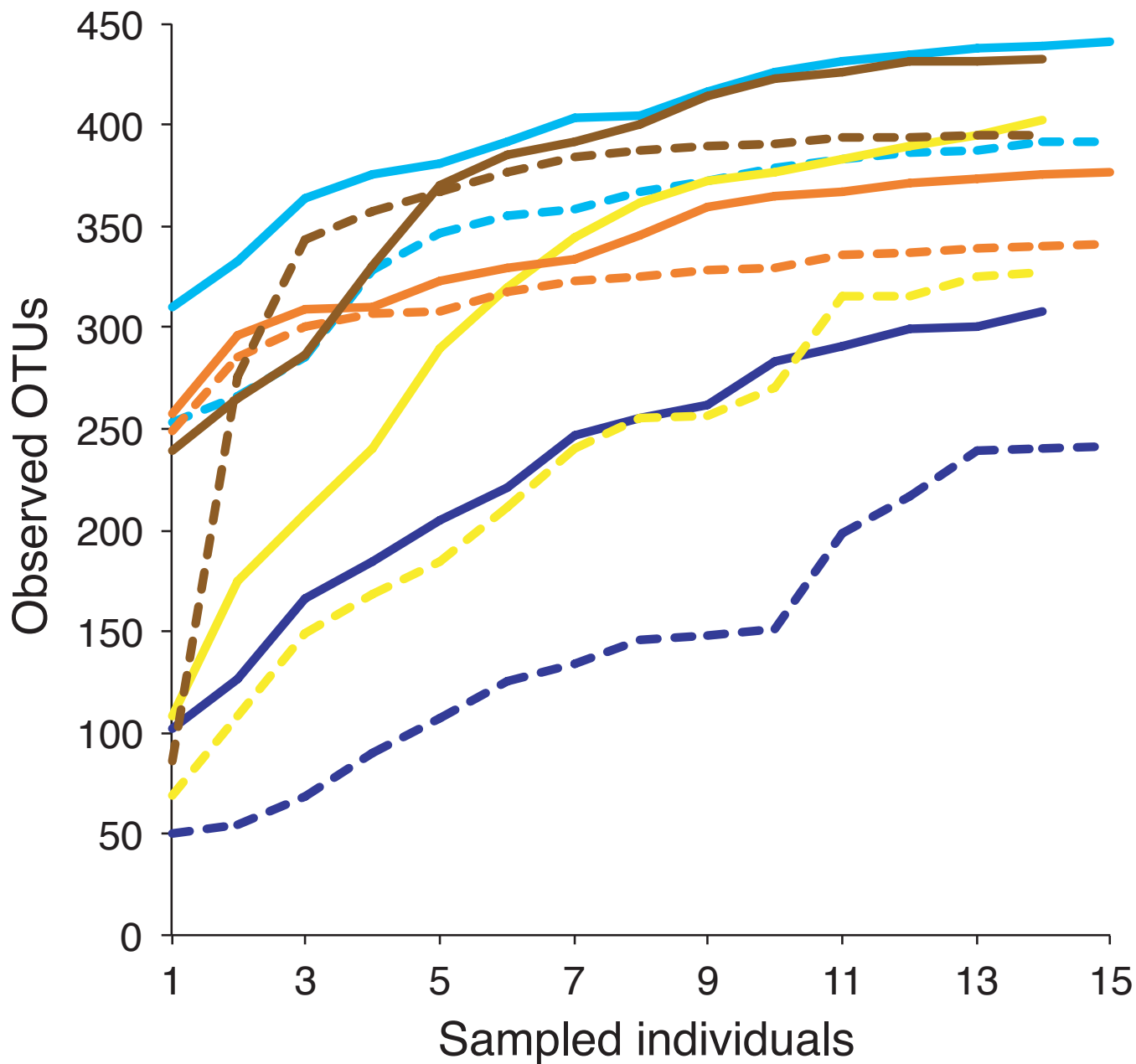

Supplement: Supplementary file 12 — OTU accumulation plots. Individual birds were added in the order they were sampled. (PDF 124 kb) [file 42523_2019_2_MOESM12_ESM.pdf]
